# Expandable Total Humeral Replacement in a Child with Osteosarcoma

**DOI:** 10.1155/2015/690159

**Published:** 2015-05-18

**Authors:** Eric R. Henderson, Jidi Gao, John Groundland, Odion Binitie, G. Douglas Letson

**Affiliations:** ^1^Sarcoma and Connective Tissue Oncology Program, Department of Orthopaedic Surgery, Norris Cotton Cancer Center, Dartmouth-Hitchcock Medical Center, The Geisel School of Medicine at Dartmouth College, One Medical Center Drive, Lebanon, NH 03756, USA; ^2^The Geisel School of Medicine at Dartmouth College, Hanover, NH 03755, USA; ^3^Department of Orthopaedic Surgery, University of South Florida, MDC 106, Tampa, FL 33612, USA; ^4^Sarcoma Division, Moffitt Cancer Center & Research Institute, 12902 Magnolia Drive, Tampa, FL 33612, USA

## Abstract

*Case*. A right-handed 8-year-old female patient presented with a conventional, high-grade osteosarcoma involving her right humerus; through-shoulder amputation was recommended. After consultation, total humerus resection with expandable, total humeral endoprosthesis reconstruction was performed with a sleeve to encourage soft-tissue ingrowth. At three-year follow-up she has received one lengthening procedure and her functional scores are excellent. *Conclusion*. Total humeral resection and replacement in the pediatric population are rare and although early reports of expandable total humeral endoprosthesis outcomes demonstrate high failure rates, this patient's success indicates that expandable total humeral replacement is a viable option.

## 1. Introduction

Osteosarcoma is the most common primary malignant tumor of bone and most commonly affects children and young adults in the second decade of life. Adjuvant chemotherapy has improved the 5-year survival rate of nonmetastatic osteosarcoma to approximately 70%. Subsequent advances in three-dimensional radiological imaging and improved implant technology have facilitated limb-preserving surgery for osteosarcoma. Limb-preserving surgery in children presents the additional challenge of accommodating growth.

We report the case of a pediatric patient diagnosed with osteosarcoma of the right humerus. Prior to presentation to our clinic, upper extremity amputation was recommended for surgical management. After review of her radiographic and staging studies and completion of her neoadjuvant treatment, an expandable proximal humeral replacement was performed. The purpose of this paper is to describe her surgical management and three-year functional and oncological outcome. The patient and her parents provided consent for this study and her rights were protected.

## 2. Presentation of the Case

A right-handed eight-year-old girl presented to her primary care physician in Iowa with 2-3 weeks of right arm pain at rest and with activity. There was no history of trauma and she localized the pain to the proximal and midshaft region of the right humerus. Radiographs revealed a blastic-appearing lesion and referral to an orthopaedic oncology surgeon was undertaken. A core needle biopsy was performed which confirmed a diagnosis of high-grade conventional osteosarcoma. Staging studies were performed including a computed tomography (CT) scan of the chest, magnetic resonance imaging (MRI) of the humerus, and whole-body Technetium-99 bone scan, which revealed no evidence of metastatic disease or Enneking Stage IIB disease. Our patient then began neoadjuvant chemotherapy and surgical planning was undertaken for a through-shoulder amputation of the right upper extremity. At this point her parents contacted our clinic to discuss limb-preserving surgery.

Upon presentation to our clinic the patient had resolution of her arm pain. She had no relevant medical, family, or social history. Physical examination revealed a well-appearing, well-nourished, and interactive girl who appeared as her stated age. Her right upper extremity showed increased girth about the proximal and midshaft region of the humerus, a well-healed biopsy incision, and no skin changes; the mass was not tender to palpation. Her right arm showed normal sensory, motor, and vascular function with full shoulder and elbow motion compared to the left upper limb. The remainder of her examination demonstrated no abnormalities.

**Figure 1:**
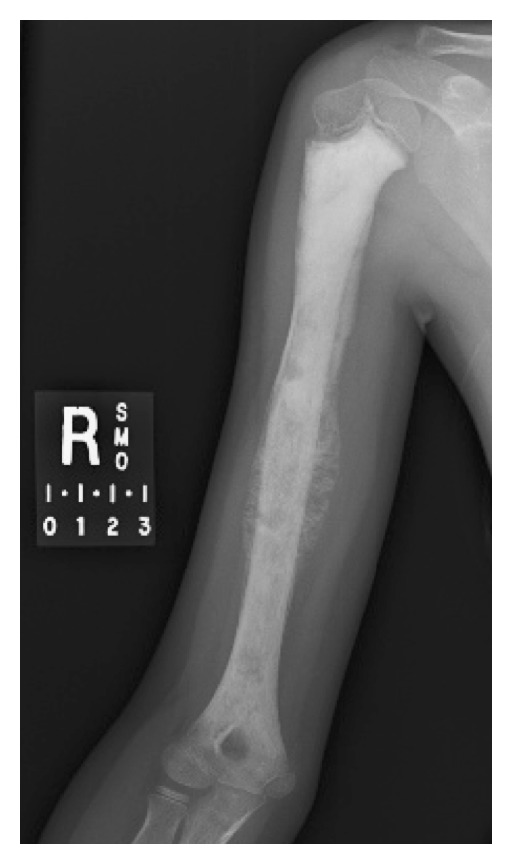
Anteroposterior radiograph of humerus following neoadjuvant chemotherapy for osteosarcoma.

**Figure 2:**
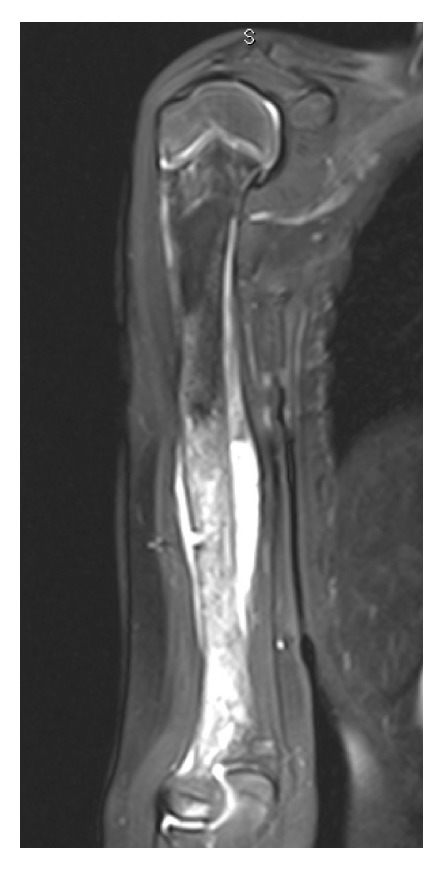
MRI of humerus demonstrating osteosarcoma lesion.

Postchemotherapy radiographs showed mineralization of her tumor (Figure 1); repeat chest CT and bone scan demonstrated no evidence of metastatic disease. MRI showed a mass occupying almost the entire humeral shaft (Figure 2).

Because local control of the tumor would require removal of the entire humerus, a total humeral replacement was recommended to the patient and her family. Due to the patient's young age and anticipated growth of 7.2 cm [1], a reconstruction which could accommodate lengthening was desired and therefore a custom, minimally invasive lengthening total humeral endoprosthesis was designed and built to her specifications by Stanmore (Stanmore Implants, Elstree, UK). The device had a lengthening capacity of 8 cm.

**Figure 3:**
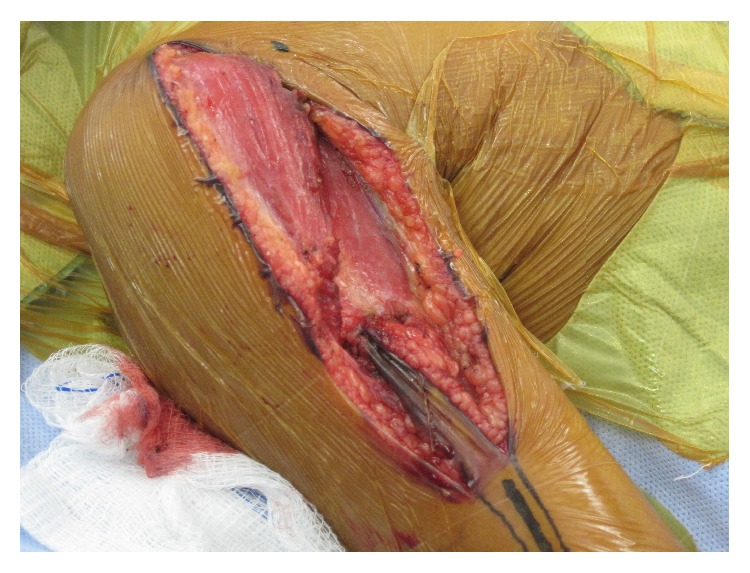
Surgical approach to the proximal humerus.

**Figure 4:**
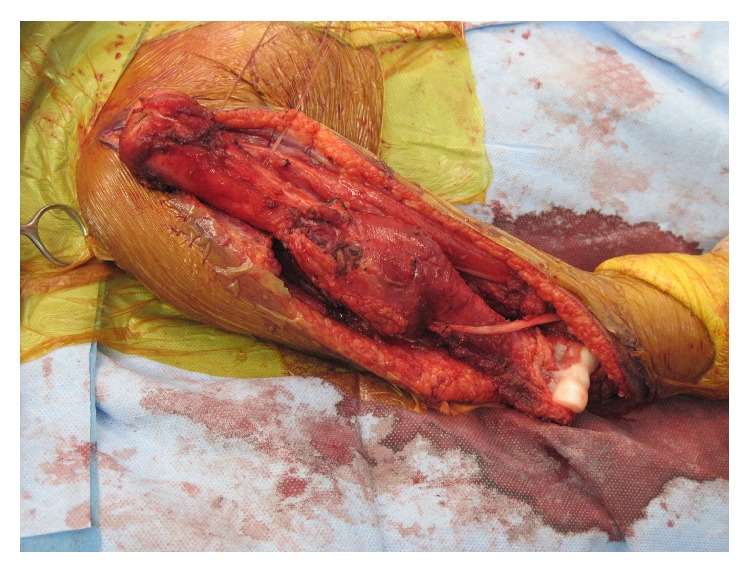
Total humeral surgical specimen following dissection.

**Figure 5:**
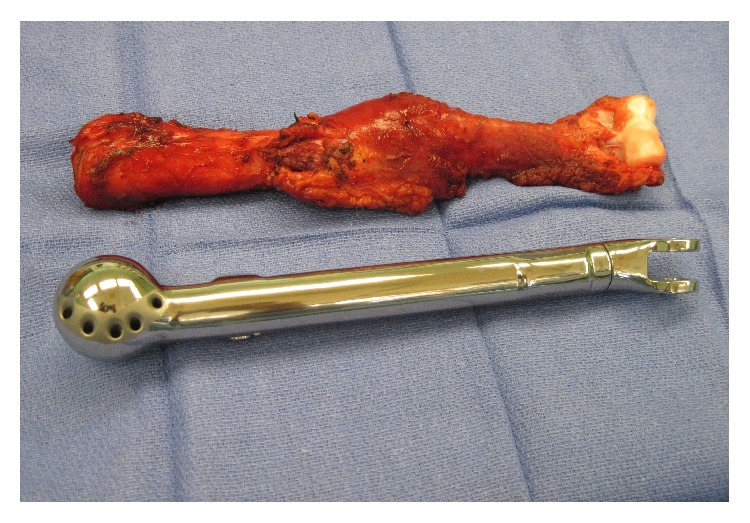
Total humeral surgical specimen and expandable total humeral endoprosthesis.

**Figure 6:**
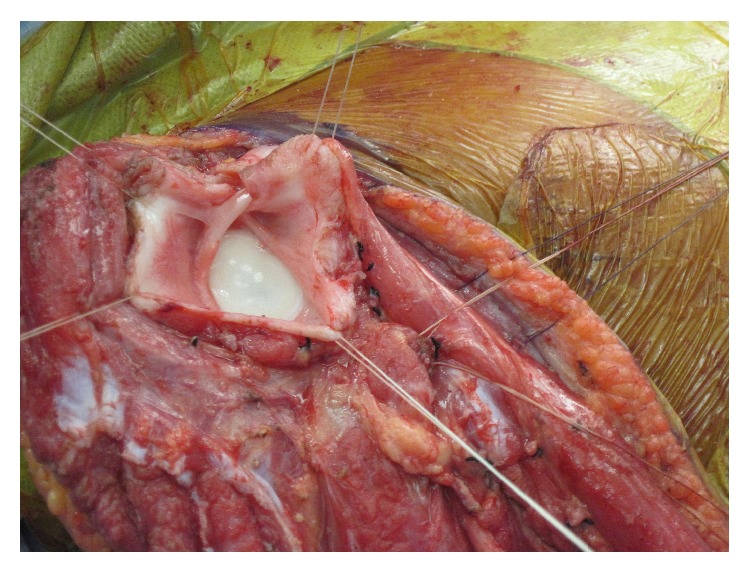
Preserved rotator cuff and shoulder joint capsule marked with tag sutures.

**Figure 7:**
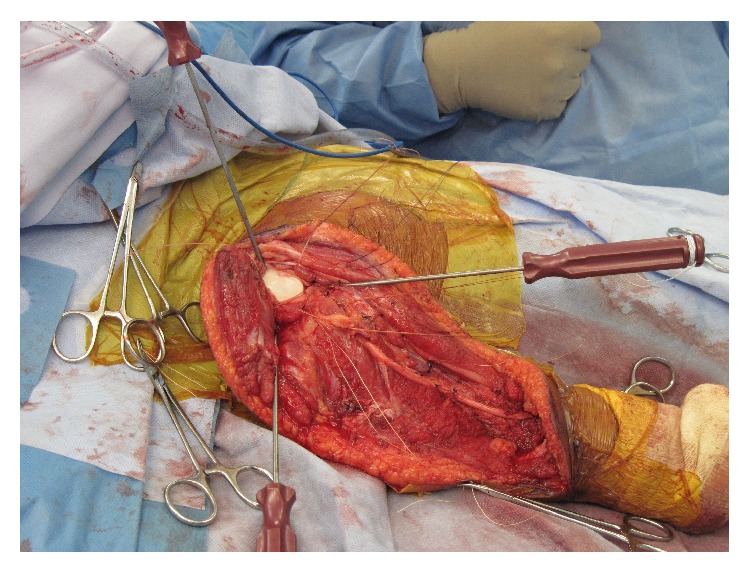
Placement of suture anchors about glenoid neck.

**Figure 8:**
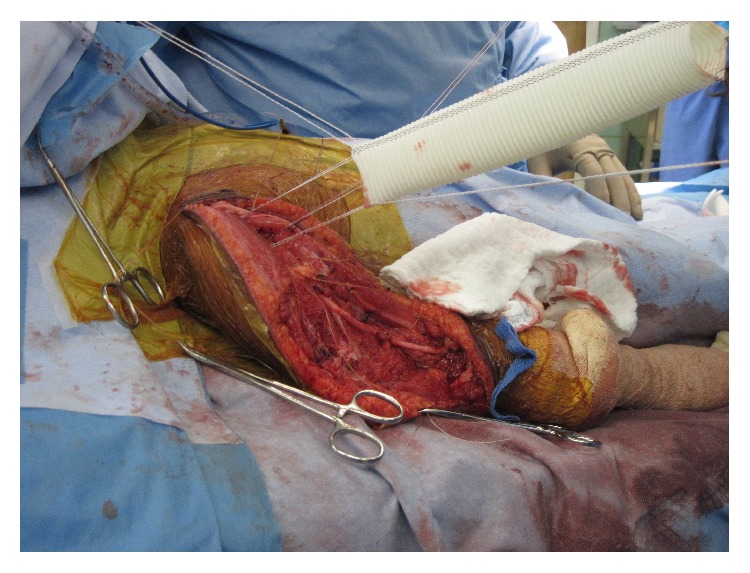
Vascular graft being positioned over glenoid prior to placement of endoprosthesis.

**Figure 9:**
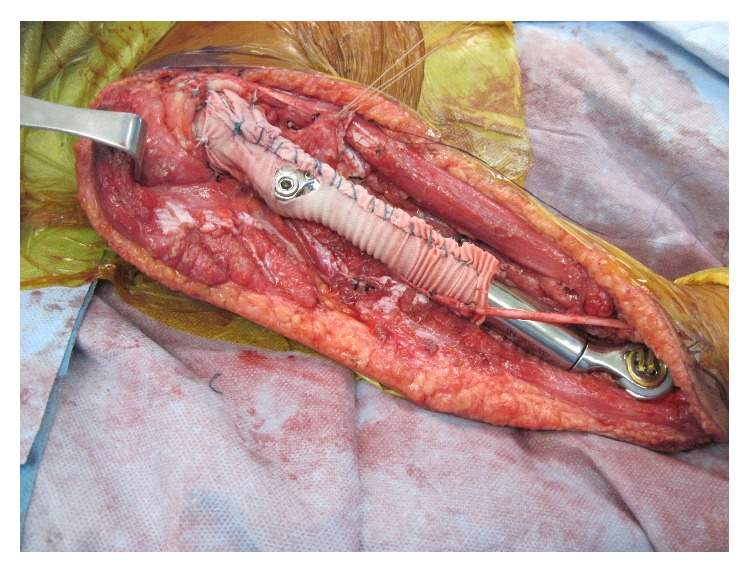
Endoprosthesis following ulnar cementation, suturing of rotator cuff to vascular graft, and imbrication of the vascular graft with nonabsorbable sutures.

**Figure 10:**
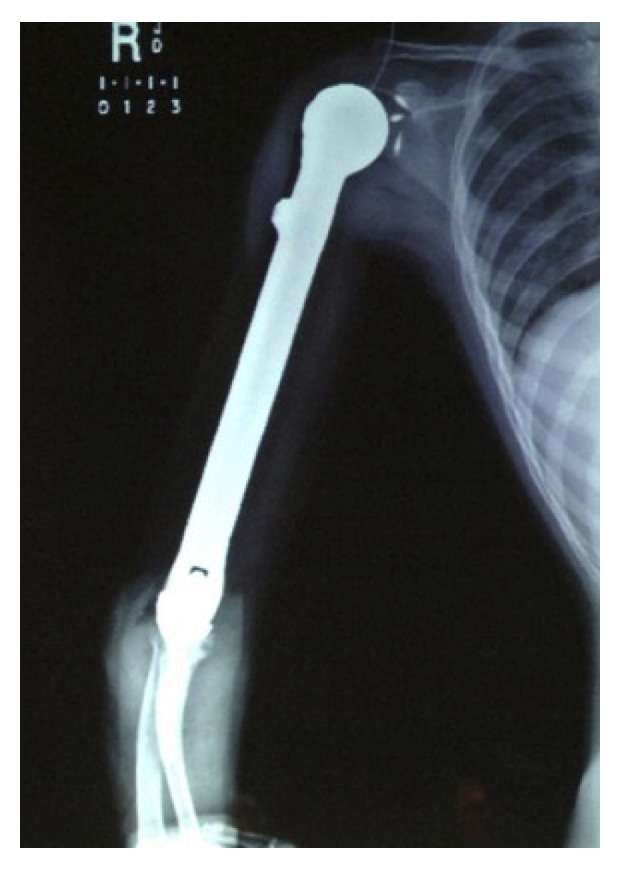
Postoperative anteroposterior humeral radiograph with implanted endoprosthesis.

Surgical resection of her tumor was performed through an extensile anterior approach to the shoulder joint, anterolateral approach to the humeral shaft, and lateral approach to the elbow joint (Figure 3). The brachialis muscle origin was left on the specimen as it covered the soft-tissue mass and the radial nerve was preserved (Figure 4). A custom, expandable endoprosthesis was manufactured prior to surgery based on measurements from preoperative imaging studies (Figure 5). The rotator cuff was dissected free of the humerus at its insertion and preserved (Figure 6). Suture anchors were inserted into the glenoid (Figure 7). A vascular graft was incorporated into the anchor sutures (Gore Industries, Flagstaff, AZ, USA) (Figure 8). The vascular graft was imbricated about the endoprosthesis with a fenestration to accommodate lengthening mechanism access (Figure 9). The humeral musculature was sewn to the vascular graft to encourage soft-tissue ingrowth. The ulnar component was cemented. The incision was then closed in layers without a drain and she was placed in a splint. At the conclusion of the operation, motor and sensory function was complete. Postoperative radiographs showed the implant to be in good position (Figure 10). In the postoperative period shoulder motion was restricted in an effort to encourage rotator cuff ingrowth into the vascular graft. Postoperative physical therapy was begun six weeks after surgery beginning with passive shoulder motion, gradually transitioning to active shoulder motion after three months.

At present our patient is three years from surgery and remains without evidence of local or distant disease progression. She has undergone one lengthening operation with a total of 0.7 cm of expansion. Her shoulder demonstrates passive abduction and flexion of 0–120 and 0–135, respectively. Active shoulder abduction and flexion are 0–110 and 0–130, respectively. Shoulder strength in both abduction and flexion are 4/5; strength with internal and external rotation is 2+/5. Active and passive elbow motion are 0–125 with strength of 3/5 in flexion and 4/5 in extension. She was able to resume playing the piano without difficulty or discomfort. Adolescent PODCI self-reported global mean and normative scores were 92 and 46; adolescent PODCI parent-reported global mean and global normative scores were 95 and 50, respectively; PODCI domain scores are reported in Table 1.

## 3. Discussion

The goal of limb-sparing surgery for connective tissue cancers is complete tumor excision leading to disease-free survival with a functional limb [2]. While limb salvage techniques have evolved substantially over the last fifty years and oncological results are comparable to amputation, the goal of complete restoration of function has not been realized and surgical complications remain significantly higher than comparable primary total joint arthroplasty procedures [3].

Endoprosthesis reconstructions with uniaxial and polyaxial articulations are affected by different failure modes; uniaxial articulations are frequently subject to aseptic loosening and polyaxial joints are commonly unstable [3]. Total humeral reconstructions are subject to failure modes relevant to both joint types and have higher failure rates than either proximal or distal humeral replacements [3].

Because the preponderance of pediatric primary malignant bone tumors occurs about the knee and hip, expandable implants were first described for use in the lower extremity by Scales et al. in 1976 [4]. Since that time, numerous authors have published small series reporting generally favorable patient outcomes for lower extremity expandable implants [5–24]. In addition to conventional modes of failure [3], expandable implants are vulnerable to problems of lengthening mechanism failure and adjacent physeal injury [24–26].

Use of expandable endoprostheses in the upper extremity is far less common than in the lower extremity, due to both the distribution of pediatric bone cancers and the difficulty of creating a lengthening mechanism that fits neatly into a pediatric humerus-sized package. An expandable humerus implant was first conceived in 1985 by the Biomechanical Engineering Department at the Royal National Orthopaedic Hospital at Stanmore. Lavy and Briggs reported on the use of the Stanmore implant for total humerus replacement and the results were poor with all three implants resulting in failure [27]. According to our literature review, expandable total humeral replacement has been attempted an additional 23 times, with over one-half of surviving patients experiencing failure of their device (Table 2). While these results suggest an abysmal forecast for patients receiving an expandable total humeral replacement, it should be noted that 21 of these patients underwent surgery in the 1980s and 1990s, when endoprosthesis design and use were still in their earliest phase. Although still a nascent technology, early failures have led to improved understanding of the mechanical challenges facing these reconstructions and will likely lead to improved outcomes as results of newer implants are reported. The authors believe the patient described here has obtained good shoulder function through a combination of the patient's young age and meticulous reconstruction of the rotator cuff using a porous in-growth surface.

To our knowledge the case presented here is the seventh expandable total humerus replacement performed in the US and is the first to combine the Stanmore minimally invasive lengthening implant with a porous sleeve for soft tissue ingrowth. Current technology does not allow for noninvasive lengthening; however, this will likely be available in the future. Revision of the current prosthesis is not mandatory and will be undertaken only in the event of failure. Based on the excellent oncological and functional results of our patient, and the antithetical alternative of upper extremity amputation, we believe that expandable total humeral replacement is an excellent option for the skeletally immature patient meeting indications.

## Figures and Tables

**Table 1:** PODCI domain scores.

	Domain											
	Upper extremity		Mobility		Sports		Pain		Happiness		Global	
	(m)	(n)	(m)	(n)	(m)	(n)	(m)	(n)	(m)	(n)	(m)	(n)
Self-reported	92	34	100	52	82	39	93	53	95	57	92	46
Parent-reported	96	44	100	52	85	42	100	57	95	57	95	57

(m): mean; (n): normative.

**Table 2:** Literature summary for expandable total humerus replacements.

Authors	Year	Implants	Surviving patients	Failures	Failure modes (n)
Lavy and Briggs [27]	1992	3	3	3	1 (2); 4 (1)
Unwin and Walker [19]	1996	7	NA	1	4 (1)
Ayoub et al.	1999	6	2	0^∗^	0^∗^
Eckardt et al. [11]	2000	5	NA	NA	NA
Young et al.	2011	1	1	0	
Natarajan et al.	2011	2	2	1	5 (1)
Puri, Gulia	2012	2	1	1	3 (1)

Failure modes: 1: soft tissue; 2: aseptic loosening; 3: structural; 4: infection; 5: disease recurrence. ^∗^One patient had a forequarter amputation for pain unrelated to the prosthesis.
